# Persistent Bacillus cereus Bacteremia in an Immunocompetent Patient With a History of Polysubstance Abuse

**DOI:** 10.7759/cureus.33650

**Published:** 2023-01-11

**Authors:** Antoine Boustany, Noor Ramahi, Joya-Rita Hindy, Marcos Garcia, K. V Gopalakrishna

**Affiliations:** 1 Internal Medicine, Cleveland Clinic Foundation, Cleveland, USA

**Keywords:** antibiotics therapy, polysubstance use disorder, gram positive bacteremia, gram-positive meningitis, bacillus cereus

## Abstract

Extraintestinal infections are rare with *Bacillus cereus* and include endocarditis, pneumonia, and meningoencephalitis. It has been primarily reported in immunosuppressed individuals with hematological malignancies and rarely in people who inject drugs (PWIDs). Herein, we report the case of a healthy adult woman with no underlying conditions except for injection drug use who presented with signs of meningitis. A 40-year-old female intravenous (IV) drug addict presented to the hospital with a chief complaint of severe headache. She had a fever of 38 °C, and her neurological examination was unremarkable. Laboratory results were significant for a white blood cell (WBC) count of 20.0 × 10^9^/L (reference range: 4.5 to 11.0 × 10^9^/L) and urine toxicology that was positive for amphetamines and cocaine. A lumbar puncture showed a total of 1,736 nucleated cells/µL, 88% neutrophils, a glucose level of 73 mg/dL, and a significantly elevated protein level of 155 mg/dL. *B. cereus *grew in blood cultures and cerebrospinal fluid (CSF) cultures. Once the growth of *B. cereus* was identified in the CSF, intravenous vancomycin was started. After leaving against medical advice (AMA), the patient presented again to the hospital, and a lumbar puncture was repeated. Cerebrospinal fluid showed total nucleated cells of 13 cells/µL, but the patient remained bacteremic. An echocardiogram, computerized tomography (CT) of the abdomen and pelvis, and tagged white blood cell scan could not identify a source for the bacteremia. Despite receiving two weeks of IV vancomycin, her blood cultures remained consistently positive for *B. cereus *without identifying a clear source of infection. Although *B. cereus* rarely affects the central nervous system, there have been a few cases where immunosuppression has been linked to the infection. We report an unusual case of a patient who continued to be bacteremic despite a thorough search for a source of *B. cereus *infection and IV vancomycin treatment. As a result, we raise the possibility of addictive behavior due to the patient's pattern of leaving the hospital against medical advice and returning with recurrent bacteremia. A thorough history and careful search for a source of infection are required when *B. cereus* grows persistently in blood cultures.

## Introduction

*Bacillus cereus* is a spore-forming Gram-positive bacillus [1] able to survive in the toughest environmental conditions. This bacterium is mainly involved in food-poisoning outbreaks associated with rice, causing self-limited gastrointestinal disease [2]. Although limited, there are some previous case reports of extraintestinal infections [3], including bacteremia [4], endocarditis [5], pneumonia [6], and meningoencephalitis [7]. *B. cereus* bacteremia has been particularly described in immunocompromised adults with hematological malignancies [8-9], and rarely in people who inject drugs (PWID) [10-11]. Herein, we report the case of a healthy adult woman with no underlying conditions except for injection drug use (IDU) who presented with signs of meningitis.

## Case presentation

A 40-year-old female with a past medical history of type 1 bipolar disorder, major depressive disorder (MDD), migraines without aura, hypothyroidism, and drug use presented to the hospital with a chief complaint of severe headache.

Her headache started one day before the presentation and was described as bilateral and generalized. It was different from her usual migraines as it was associated with photophobia and nausea that lasted for hours. She reported subjective fever, shortness of breath, generalized weakness, and myalgia. The patient denied having neck pain, chills, or urinary, pulmonary, or gastrointestinal symptoms. She also denied exposure from sick contact, recent travels, eating uncooked meat, drinking contaminated water, and swimming in lakes. She was unsure if she was up to date with her routine vaccinations. She is a chronic smoker (one pack a day) and does not drink alcohol. As for her drug use history, the patient is an intravenous (IV) heroin addict and a crack cocaine abuser. She mentioned that the day prior to admission, she used bath salts.

On presentation, the patient was hemodynamically stable and oxygenating well on room air. She had a fever of 38 °C. On physical examination, the patient was comfortable, and her cardiac, respiratory, and abdominal examinations were unremarkable. Neurologic examination showed minimal nuchal rigidity, most likely muscular in nature, with normal cranial nerves, cerebellar, and ophthalmologic examinations. Motor power and sensation in the extremities were preserved. The signs from Kernig and Brudzinski were negative. Skin examination revealed multiple lesions and scratches on her right forearm, consistent with the site of IV drug insertion and without evidence of infection or abscess. Laboratory results were unremarkable except for leukocytosis with a white blood cell count (WBC) of 20.0 × 10^9^/L (reference range: 4.5 to 11.0 × 10^9^/L) and a urine toxicology test that was positive for amphetamines and cocaine metabolites. Lumbar puncture revealed 88% neutrophils (reference range: 0-3%), 3% lymphocytes (reference range: 50-90%), red blood cells (RBC) of 206 cells/µL (reference range: 0-5 cells/µL), a glucose level of 73 mg/dL (reference range: 40-70 mg/dL), total nucleated cells of 1,736 cells/µL (reference range: 0-5 cells/µL), and a significantly elevated protein level of 155 mg/dL (reference range: 15-45 mg/dL). Cerebrospinal fluid (CSF) and blood cultures reported growing *B. cereus* that was susceptible to meropenem, vancomycin, and gentamycin, as represented in Table 1. A computerized tomography (CT) of the brain without IV contrast was negative for acute intracranial abnormalities. Therefore, a magnetic resonance imaging (MRI) with and without IV contrast was done and showed findings suggestive of intracranial hypertension, including flattening of the pituitary, prominence of the bilateral optic nerve sheaths, and suspected narrowing of the bilateral transverse sinuses. These findings were consistent with the post-lumbar puncture effect. The patient was started on intravenous vancomycin for bacterial meningitis complicated by bacteremia, due to *B. cereus*. Two days after her admission, the patient decided to leave against medical advice (AMA) before completing her course of antibiotic treatment.

**Table 1:** The different antibiotic MIC levels and susceptibilities of Bacillus cereus MIC: minimum inhibitory concentration

Antibiotic type	MIC	Susceptibility
Penicillin	≥16	Resistant
Meropenem	0.23	Susceptible
Ceftriaxone	≥4	None
Vancomycin	0.5	Susceptible
Gentamycin	≤2	Susceptible

The next day, the patient presented again to the emergency department with complaints similar to those of her first admission. She was admitted to the regular nursing floor to resume her antibiotic course and for further workup. Despite physical examination and imaging not being indicative of meningitis, the suspicion remained high in the setting of elevated protein and total nucleated cells in the CSF. Therefore, lumbar puncture was repeated, revealing total nucleated cells of 13 cells/µL (reference range: 0-5 cells/dL). The protein level normalized to 39 mg/dL (reference range: 15-45 md/dL) and there was no growth of any organisms in the second CSF culture. However, the patient remained bacteremic, and further evaluation was initiated to identify the source of the infection. An echocardiogram was performed to rule out endocarditis and showed a left ventricular ejection fraction of 70% (reference range: 50-70%) with normal valve leaflets and without associated mobile echodensities. Subsequently, a transesophageal echocardiogram (TEE) was ordered, and its results were similar to the transthoracic echocardiogram. A CT of the abdomen and pelvis with IV contrast was also performed and showed a ventral hernia in the lower abdomen without evidence of an intraabdominal infection or abscess. To evaluate for possible cerebritis or ventriculitis, an MRI of the brain was done with and without IV contrast and came back negative for any acute pathologies. Unfortunately, before completion of the workup, she left AMA a second time for unclear reasons.

The patient came back again the following day with non-resolving symptoms. A tagged WBC scan was performed and showed a mild labeled WBC accumulation in the bilateral pelvic and inguinal lymph nodes, representing a reactive process. There was no other evidence suggesting an acute inflammatory or infectious process. A PICC line was placed, and the patient was discharged to the skilled nursing facility with instructions to continue IV vancomycin and remove the line once her blood cultures turn negative.

## Discussion

**Figure 1:**
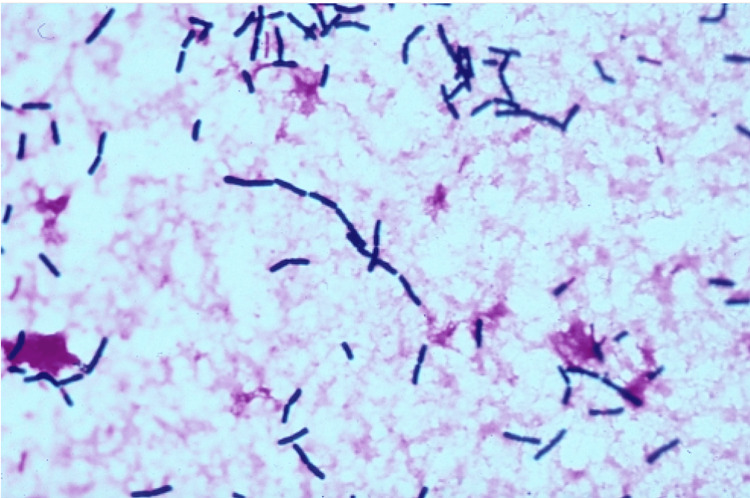
Gram stain of blood culture showing Gram-positive slender bacilli with rounded ends singly, in pairs, and in short chains

*Bacillus cereus* is a Gram-positive, aerobic or facultatively anaerobic, rod-shaped, motile, spore-forming bacteria (Figure 1) [1]. The Centers for Disease Control (CDC) reports that there were 19,119 outbreaks overall and 37,531 illnesses related to Bacillus-related illnesses from 1998 to 2015. Of these, there were 14,681 hospitalizations and 337 deaths during those years. These refer to all Bacillus-related illnesses, not just *B. cereus*-related illnesses. Specifically, there are an estimated 63,400 episodes of *B. cereus* illness annually in the United States. Although everyone is susceptible to *B. cereus* infection, mortality related to this illness is uncommon. *B. cereus* is known to cause a self-limiting gastrointestinal emetic or diarrheal syndrome from the toxins it produces. These toxins include emetic toxin and enterotoxin; however, there are other toxins produced by this bacterium that can contribute to the pathogenicity of *B. cereus* in non-gastrointestinal diseases. These include phospholipases, proteases, and hemolysins [11]. These tissue-destroying exoenzymes are increasingly being reported as the cause of serious and potentially fatal non-gastrointestinal infections. The emetic enterotoxin has been associated with a few cases of liver failure and death in otherwise healthy people [10].

Central nervous system involvement with *B. cereus* is rare, but there have been a few reported associations with immunosuppression [12]. Bacteremia with Bacillus species (spp.) has been published in the literature, mostly in immunocompromised hosts. Specifically, hematologic patients have a higher risk for *B. cereus* bacteremia and invasive *B. cereus* infection. This is thought to be due to neutropenia and skin barrier exposure from central venous catheters and after high-dose chemotherapy [12]. *B. cereus* is also associated with infections involving the cardiovascular, central nervous, respiratory, ocular, and musculoskeletal systems in intravenous drug users. Such infections include cellulitis, endocarditis, and panophthalmitis. Even in these cases, with prompt antibiotics, the prognosis is good. Morbidity and mortality rates are higher in populations with prosthetic valve endocarditis who require valve replacement [10]. It is important to note that Bacillus spp. are common lab contaminants, but multiple sets of blood cultures do indicate infection [13]. There have been reported findings of heroin laced with *B. cereus* when it was being grown. It was found to be a direct contaminant [10]. What this tells us is that *B. cereus* is often a contaminant in heroin, which can lead to higher rates of infection in intravenous drug users.

The novelty, in this case, is extensive imaging, and the lab did not find a source of infection in an IV drug user who had consistent positive blood cultures for *B. cereus*. Our patient’s CSF cultures came back negative; however, the protein in the CSF was elevated, raising concern for true meningitis. This was a puzzling piece as a second lumbar puncture came back negative for meningitis and exhaustive imaging; the physical exam and labs were normal, although she was consistently bacteremic with blood cultures on IV vancomycin. Physical exams by multiple providers did not yield a source either. Our patient, throughout the course of her hospital stay, did not look like the typical meningitic or bacteremic patient. On the contrary, she looked healthy.

Therefore, we raise the suspicion of addictive behavior as our patient kept leaving against medical advice and returning with persistent bacteremia on IV antibiotics. It may be that our patient injected herself more than once with a substance containing the organisms, as she was persistently bacteremic, even though the bacteria were cleared from the CSF and the MRI of the brain was negative for ventriculitis. Although we were not able to find the source of the infection, it is likely a compound containing Bacillus spores. The patient did admit to having dirty water at home from a bad septic tank, which could have been the source [14]. Persistent blood cultures growing *B. cereus* in an immunocompetent host with no source of infection in the workup warrant a detailed history from the patients.

## Conclusions

Our report emphasizes the importance of a thorough history and vigilance for non-gastrointestinal infections caused by *B. cereus*. We report an unusual case of a patient who continued to be bacteremic despite a thorough search for a source of *B. cereus* infection and IV vancomycin treatment. Persistent blood cultures positive for *B. cereus* in an immunocompetent host should not be considered as contaminant without accurate evaluation as it can represent an underlying occult infection. Despite an extensive workup and treatment with IV vancomycin, the patient remained bacteremic. As a result, we raise the possibility of addictive behavior due to the patient's pattern of leaving the hospital against medical advice and returning with recurrent bacteremia. Although in this present case we were not able to find the source of the infection, it could be a compound containing Bacillus spores. Obtaining a psychiatric history helps with diagnosis and may prevent additional infections and complications.
